# Analysis and Strategies of Internet + Taxation Risk Management of Listed Companies in the Big Data Era From the Organizational Psychology Perspective

**DOI:** 10.3389/fpsyg.2022.904417

**Published:** 2022-06-24

**Authors:** Xuan Zhao

**Affiliations:** ^1^School of Public Finance and Administration, Harbin University of Commerce, Harbin, China; ^2^Department of Management, Harbin Finance University, Harbin, China

**Keywords:** fiscal and taxation risk management, listed companies, Internet +, big data, risk identification

## Abstract

The massive amount of information brought about by the era of big data has enormous potential value. In-depth discussion and analysis of solving the information asymmetry between tax collection and taxpayers are keys. This paper provides an in-depth study and analysis of the fiscal and tax intelligent risk management strategies of listed companies in the big data environment. The tax risk management of listed companies is optimized. Tax authorities should follow the development trend of big data, apply big data thinking, comprehensively excavate and utilize tax-related information, and improve the tax risk management model. At the same time, they should make full use of information technology to solve the problem of information asymmetry between tax collection and taxpayers and improve the quality and efficiency of tax risk management. The work discusses the problems of the current tax collection and management model in terms of management thinking, information collection, data analysis, legal system, and talent technology, and analyses the challenges brought by big data technology to the tax collection and management model as well as tax source management and tax informatization.

## Introduction

By using big data technology, scattered data can be integrated and analyzed to promote the efficient development of various tasks, and this process has occurred in just a decade. Based on the development of the Internet, the new economy develops rapidly, breaking the traditional “flow of things” characterized by the realization of value, and replacing it with a personalized modern service based on data collection and feedback to match demand and enterprise supply (Moşteanu and Faccia, 2020). The industrial scale and the tax revenue of listed pharmaceutical manufacturing enterprises has grown rapidly. An increase in the diversity and complexity of production and operation has been brought about by this expansion in scale, and by the special circumstances and unwritten rules unique to the industry. This has made the industry less tax compliant, making its tax risks diversified, complex, and highly concealed such that traditional single account tax risk analysis can no longer meet the needs of tax risk identification for listed companies (Gong and Janssen, 2021). In recent years, the changes in national drug management impacted on the industry business model of existing listed companies. The tax risk of listed companies will also undergo new changes under the change of business model, so new requirements can be put forward for the tax risk management of this industry. Risk management has evolved to become a very promising industry.

According to the background of the rapid development of the listed company industry and the increasing economic volume, although listed companies are not the pillars of China's economy, they have developed into an important part of the national economy and become the key industry in manufacturing tax collection and management (Mikuriya et al., 2020). We aggregate the development direction of the industry and provide a reference for strengthening the quality and efficiency of tax collection and management through the study of tax risk management in the pharmaceutical manufacturing industry. From the research content, with the continuous progress of big data technology, the golden three tax system will continue to be improved and upgraded, so that tax authorities have the conditions and opportunities to obtain and use more comprehensive, complete, and systematic data at a more in-depth level (Barbeito-Caamaño and Chalmeta, 2020). By using literature aggregation construction of a big data risk identification model, and example verification, we have completely realized the optimal reconstruction of key aspects in tax risk management of listed companies and solved the main problems existing at the present stage. Financial crisis warning is a branch of financial risk research, which is crucial for every industry. For example, construction models are usually used at home and abroad to analyze the overall listed company or one of the industries, but previous studies applied to a specific new energy company have not been available. The financial characteristics of each industry are different, and there are also subtle differences in the financial characteristics of companies in the same industry (Zhang and Srite, 2021).

Early warning of a financial crisis comes from analysis of the financial risks that the enterprise may face, monitoring, and alerts, and plays an important role in the timely prevention and control of a financial crisis. For the enterprise, not being aware of a possible financial crisis or not having an accurate understanding of financial crises may have unimaginable consequences. The study of financial and non-financial indicators can make an early warning model more accurate and precise. Logistic models are established by using many indicators of listed new energy companies while using big data as the background to make the models more accurate. The logistic model can solve the problem that using only financial indicators will have a greater impact on the objectivity and comprehensiveness of the financial crisis early warning model. The introduction of the background of big data makes the rapid development of technology into the model, which is also increasing the influence of non-financial indicators on the model, thus, improving its accuracy, and making the crisis early warning model applicable to the new energy company. It is also possible to predict the financial risk of the company.

## Current Status of Research

Global scholars' research on electronic information technology in tax collection and administration originated in the middle of the last century (Hwang et al., 2021). The United States has already introduced computers into tax collection and administration, which has improved the ability and quality of tax collection; a decade later, the United Kingdom and Japan also gradually applied information technology to tax collection and administration, and the efficiency of their tax officers has been improved, and the accuracy of tax-related information has been enhanced (Meng and Cheng, 2020). In a study, Kalogiannidis pointed out that in tax source management, information technology construction is a crucial link (Kalogiannidis, 2021). Information technology construction is a way to process tax-related information based on computer and Internet technology, which enables tax-related information that cannot be processed manually to be stored and processed at high speed through information technology. To grasp the tax source situation comprehensively and objectively, the promotion of information technology construction also has a positive impact on the tax source management of each article. Göbel pointed out that Internet resources are a good basis for tax informatization, and tax informatization should be realized utilizing the Internet (Göbel and Li, 2021). Most people think that tax source management is insignificant, the textbooks on public finance also lack this aspect, so people rarely conduct in-depth research on tax source management (Tech, 2020). But taxation emphasizes feasibility, and an unmanageable tax system will lose its value. A tax system that is perfect in theory, but whose intentions are distorted in practice will be deficient (Ahn and Sura, 2020; Hewitt and White, 2021; Wu et al., 2021).

Accessing big data on tax can help build an intelligent government. Achim et al. emphasize that reliance on information technology can transform the information society into an intelligent society and aim to build an open and intelligent government that can liaise across departments through e-government (Achim et al., 2021). Chu et al. discussed the issue of government information in the era of big data and point out that the main source of big data for taxation is public sector information (Chu et al., 2020). Hong et al. emphasized the advantages of using big data to analyze citizen behavior and understand services and policies (Hong and Xu, 2021). Alecto et al. emphasized the use of big data to analyze citizen behavior and understand the advantages and disadvantages of services and policies, and he saw big data as a tool to enhance mutual understanding between government and citizens (Auksztol and Chomuszko, 2020). As a modern technology, big data first emerged and was used in practice before its great value was discovered. As a result, it was mainly technology companies or research institutions that first began to study big data, and most of the understanding of big data was from the materiality and technology perspective (Leogrande et al., 2020). At present, although there is no consensus on Big Data abroad, the direction of understanding and using it is the same.

This paper summarizes the relationship between big data and informatization and points out that the development and application of informatization accelerate the generation of big data. Big data is an advanced application of informatization to the current stage of development. Firstly, informatization makes all aspects of society data-oriented, and the promotion of the Internet and computers leads to a world of data in the real world, thus generating a large amount of data of value. Secondly, big data are an advanced application of information technology at this stage, and there is a natural unity between the two. Applying the new technology of big data and the new thinking of big data to reality will generate great data value. The new technology, new concept, and new operational mode contained in big data are not static but keep changing with time. Although the use of big data technology is not mature enough now, it is an inevitable choice for tax information construction. The real data of tax sources are difficult to obtain, the process of tax source management and shared use is not perfect, the utilization rate of tax source data resources is not high, and there are hidden dangers in the security of tax source data, and so on. Analysis highlights the following reasons: the subjects and objects of tax source management do not pay enough attention; the system and process of tax source management are not sound; the technical means of tax source management do not provide enough support; and the quality of relevant personnel involved in tax source management is not enough.

## Analysis of Intelligent Risk Management Strategies for Fiscal and Taxation of Listed Companies Under Big Data

### Design of Tax Intelligent Risk Management Strategy for Big Data

At present, big data technology is developing at a high speed. The taxation authorities have started to build the corresponding information technology platform according to the design idea of big data tax administration, laying the foundation for the next step of in-depth big data tax administration. Since the launch of the Golden Three System, a large centralization of national tax data has been realized, making the data within the system complete. Since the issuance of the Action Plan for Promoting the Development of Big Data, various government departments have started to establish information-sharing guarantee mechanisms it, and information cooperation among government departments has been strengthened year by year, providing strong support for tax risk management and allowing the scope of access to third-party information to be expanded year by year (Tian, 2020). From the perspective of tax risk management, it is far from enough to rely on the return data alone for tax-related analysis of an enterprise and industry. The main aspect of information asymmetry of tax risk management is that the financial information of taxpayers held by tax authorities only comes from various types of declaration information, with less authority to collect financial data of taxpayers and data involving specific business operations, and the real reflection of the actual operation of enterprises. The internal data of enterprises and the data of financial institutions that are closely related to the operation cannot be obtained, which cannot meet the need for big data tax risk management.

The Taxation Bureau has completed the development of the enterprise financial electronic ledger collection and analysis system. However, due to the lack of legal support, it can only collect the electronic ledger sets of a few key enterprises through consultation for tax-related analysis of a single household, but cannot analyze industry financial data, resulting in the lack of data basis required for correlation analysis in the existing risk identification analysis. It is difficult to carry out data collection work outside government departments, mainly because the corresponding legal framework is relatively lagging, making data collection lack legal protection (Indrayana et al., 2020). Although it is stipulated in the Tax Collection and Administration Law that all relevant departments and units should support and assist taxation authorities in performing their duties according to law, there are no specific legal obligations and responsibilities for external data collection, and the State Administration of Taxation is not authorized to formulate relevant working systems and norms for external data collection. Only some provincial, municipal, and county governments have promulgated and implemented local tax collection and management guarantee measures, which have not yet formed legal provisions, and the lagging policies have to a certain extent restricted the development of information tax management in the era of big data. There is no legal obligation and responsibility for tax-related information exchange, so the external data collection of tax risk management will inevitably encounter many problems in the implementation process (Han et al., 2021; Ng et al., 2021; Yang et al., 2022).

**Figure 1:**
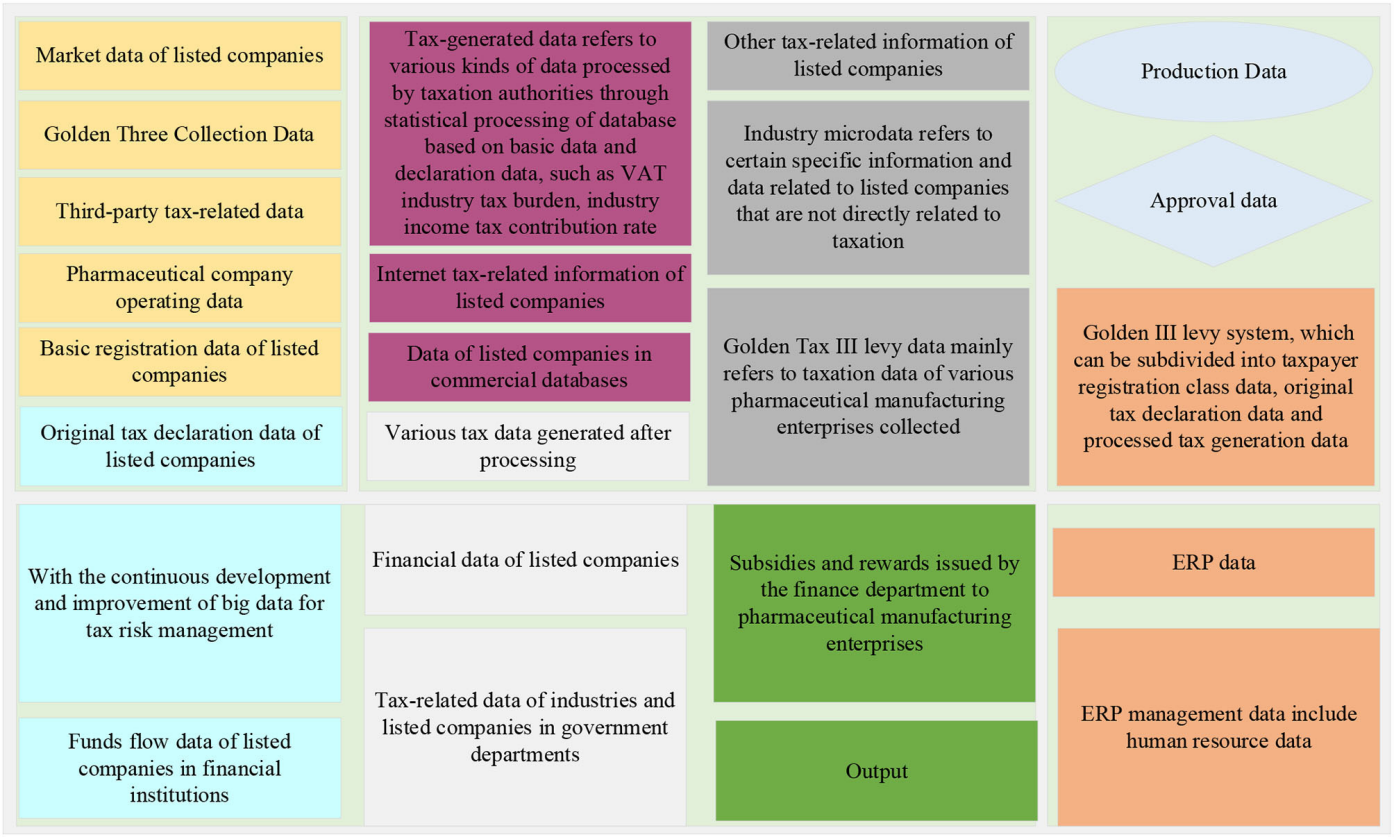
Classification of listed companies' financial taxation big data.

The tax big data of listed companies can be classified into the following categories according to their sources and their roles in tax risk management: industry market data, Golden III collection and management data, third-party tax-related data, and taxpayer operation data, as shown in Figure 1.

Macro data mainly refer to the industry macroeconomic data related to list companies released by statistical departments at all levels. Industry microdata refers to certain specific information and data related to listed companies that are not directly related to taxation released by pharmaceutical and health departments, price bureaus, and other institutions, then, Golden Tax III levy data mainly refers to taxation data of various pharmaceutical manufacturing enterprises collected and processed and generated through Golden III levy system, which can be subdivided into taxpayer registration class data, original tax declaration data and processed tax generation data. The basic registration data of taxpayers include tax registration data, identification data, etc. The original tax declaration data include various kinds of return data, financial statement data, special declaration data, etc. Tax-generated data refers to various kinds of data processed by taxation authorities through statistical processing of database based on basic data and declaration data, such as value-added tax (VAT) industry tax burden, industry income tax contribution rate, and growth rate of each tax type for each sub-sector of the pharmaceutical manufacturing industry in the current year (De Oliveira et al., 2020). The Golden Tax III collection and management data are the most basic source of big data in current risk management.

**Figure 2:**
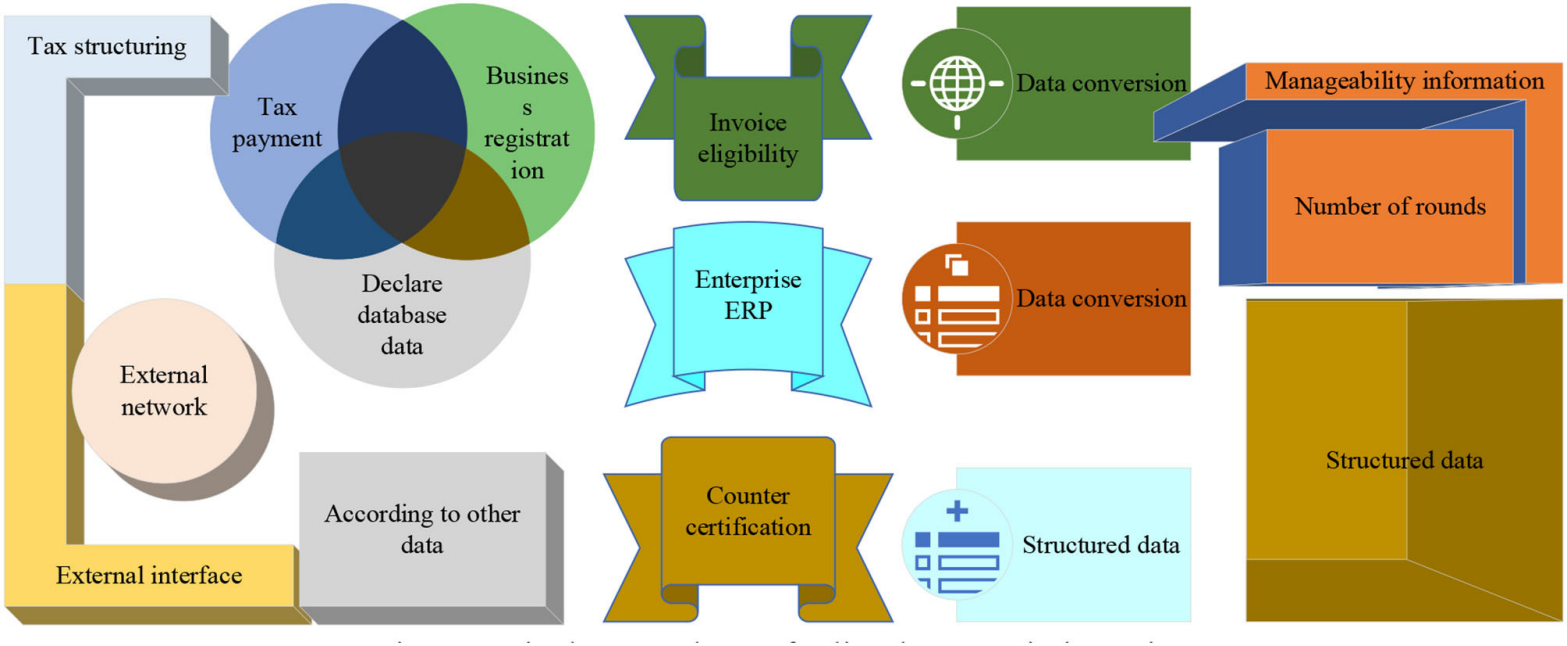
Big data warehouse for listed companies' taxation.

The third-party tax-related data refers to the tax-related data from all parties except the tax authorities and taxpayers, including the relevant tax-related data of government departments, the data of financial institutions on the capital flow of pharmaceutical enterprises, the data of pharmaceutical enterprises in the commercial database, and the tax-related information of network pharmaceutical enterprises. For example, subsidies and rewards issued by the finance department to pharmaceutical manufacturing enterprises, and the filing of R&D projects applied by enterprises in the science and technology department. With the continuous development and improvement of big data for tax risk management, the role played by obtaining third-party tax data to reduce information asymmetry in tax risk management will become increasingly prominent. The operational data of listed companies include financial data, approval class data, drug production data, and Enterprise Resource Planning (ERP) data. Financial data are mainly financial book data and vouchers and attached data, schedules of various accounting statements, related transactions, etc.; production and operational data of pharmaceutical manufacturing enterprises include production process, product, and by-product information, production, packaging, and sales records required by Good Manufacturing Practice (GMP), drug licensing information, various types of identification information, etc.; ERP management data include human resource data, production management data, procurement data, sales data, etc., as shown in Figure 2.

Precise identification of tax service objects and precise analysis of needs and characteristics is the starting point and premise of precise tax service. Without taking taxpayers' needs as a guide, precise tax service cannot be realized and can only be a baseless fantasy. Only through efficient identification of taxpayers' demands and characteristics can we realize efficient docking of tax service supply and demand. Only through accurate identification can tax service measures be more targeted and effective. As the demand for tax service gradually shows the characteristics of personalization, wisdom, efficiency, and security, for tax service to be accepted by taxpayers smoothly, precise identification of demand must be the basis, i.e., to identify and predict the demand by using big data analysis technology and analytical methods for various taxpayers, to arrive at the demand and characteristics of each precise taxpayer, and to precisely implement measures.

Precise management is an important guarantee for precise tax services. Precise tax service requires that service management must be precise, to efficiently provide matching resources for each line of service. Precision management has a role that cannot be ignored and must be used as a guarantee for orderly and efficient conveyance and the continuous improvement of the degree of overall precision. Precision management means taking a series of initiatives to control and manage the whole process of tax service, which exists in all the links of tax service precision. Big data technology and real-time data collection provide a reliable basis for tax service management decisions.

Precise assessment is a necessary means to realize precise tax services. From the viewpoint of the subject, the precision of assessment must have diversified assessment subjects. From the viewpoint of indicators, the basis of precision assessment should be more objective and scientific indicators set up beforehand. From the viewpoint of content, precision assessment requires the evaluation of the whole tax service process and its effect. Through the precise assessment and scientific application of assessment results, the feedback results should be concerned, and the subsequent tax services should be provided. It is the core of precise assessment to evaluate whether the tax service is based on the needs of taxpayers identified earlier and whether the effective and precise docking of supply and demand has been realized. To achieve good results in precise tax services, the index system should be designed with taxpayers needs as the guide. The quantitative analysis of big data simplifies the data collection and transmission so that the assessment indexes of all parties in the tax service can be truly reflected, which assesses tax service moves from subjective perception to objective evaluation, thus, realizing accurate assessment.

Precise identification, precise supply, precise management, and precise assessment from the whole process of precise tax service as four independent lines of precise tax service, which are, at the same time, closely related and organically integrated. Precise tax service is a dynamic and continuous improvement process, which helps optimize the allocation of tax service resources, improve the quality and effect of service, and alleviate the contradiction between supply and demand for tax service.

### Analysis of Intelligent Risk Management Algorithms for Fiscal Taxes of Listing Companies

Big data information is mainly extracted from the network. The financial status of the enterprise is closely related to the information characterized by the network. All external reviews can reflect the financial status and business condition of the enterprise to some extent. Meanwhile, according to signal theory, all reviews release a good or bad signal to the stakeholders. According to the results of previous studies, indicators of big data can be obtained from the following three sources: first, comments posted by users on online platforms such as forums, postings, and microblog that reflect personal emotions; second, online news and company announcements; and third, search trends reflected by search engines (Barrett, 2021). By analyzing, extracting, and studying the data in the three source channels of big data, it can be concluded that the regularity of the data of the first type of information is insufficient, and its language is rather random and emotional, so relevant judging standards and extraction and regularization techniques are needed. The second type of information is poorly correlated with the target data, and the emotion is more neutral compared with the other two, so it is not conducive to the extraction and transformation of language-specific features. The third data is poorly correlated and has great ambiguity, so it is susceptible to short-term uncertainties and is more volatile.

**Figure 3:**
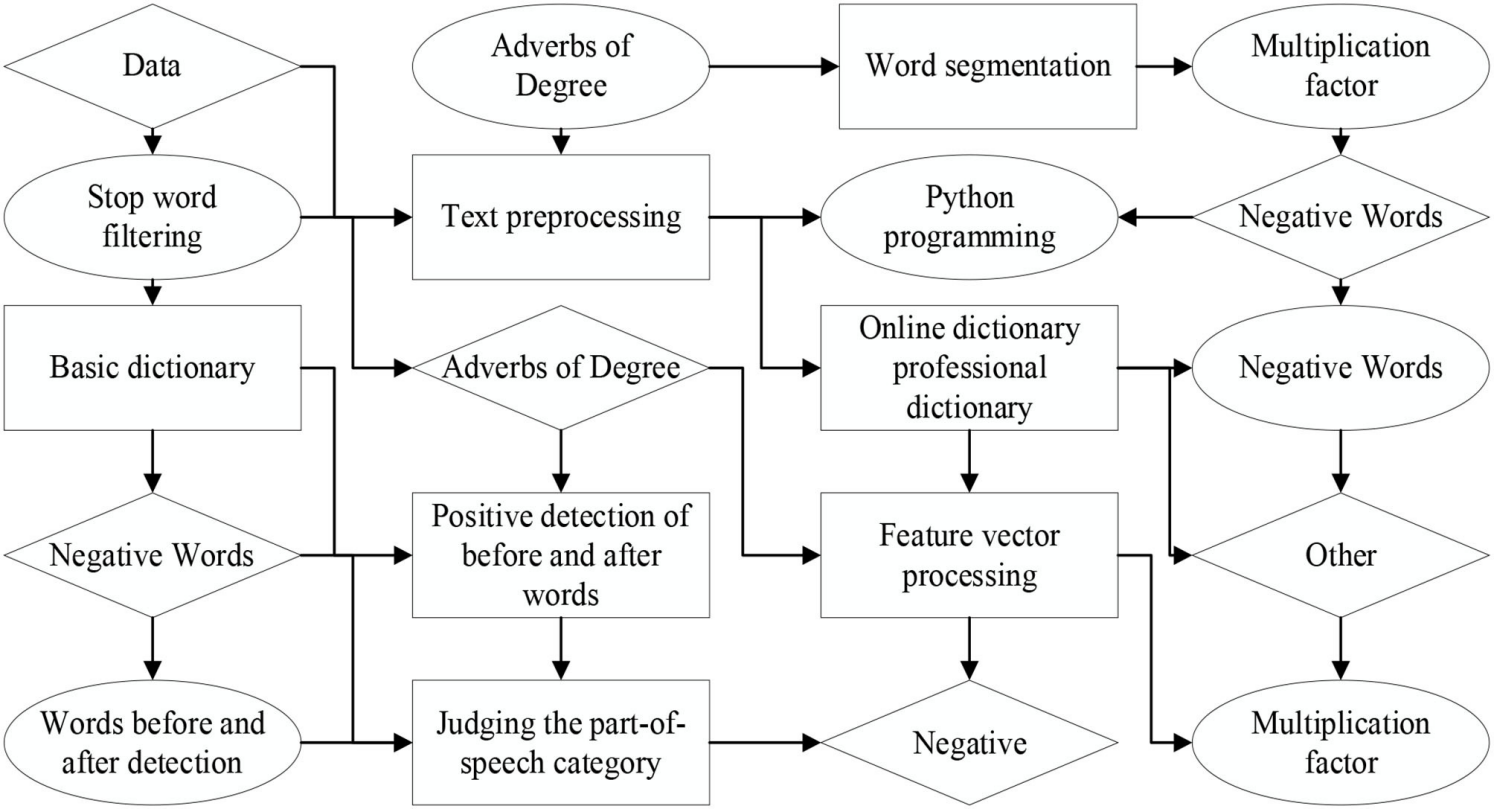
Text analysis principal flow chart.

Firstly, the construction of a big data emotional information language database draws on the natural linguistics of the language database research results. The language database is mainly divided into domain dictionaries, network dictionaries, basic dictionaries, analysis through text extraction, language cut, word frequency extraction, and language database for comparative analysis system discrimination. Secondly, analysis of big data indicators. In this paper, sentiment analysis and hotness analysis are performed for each comment individual's utterance, i.e., the analysis granularity is sentenced. The sentence-level analysis is obtained based on the average and frequency of sentiment words, leading to the number of positive, neutral, and negative comment utterances for that sample in that year. Finally, the sentiment tendency of that year is statistically analyzed, and the information of the sample is statistically derived from the number of comments and searches for each sample (Kim et al., 2021). The crawler code is written according to the specific target for target collection. The mature comment collection software is selected. Python is used as the programming basis to crawl key fields, comment titles, time, and other information calculate the number of pages and content format of the comment list, determine the collection format and order, calculate the number of comments per page and get the URL of the comment content to determine the specific object of the collection, enter the URL and collect the comment. The data format is standardized and output, as shown in Figure 3.

According to previous research and analysis, this paper combined with the characteristics of the new energy industry initially identified 56 financial indicators, but not every financial indicator can have a significantly larger impact on the financial crisis. Therefore, it is necessary to test the significance of these 56 indicators, to reduce the error interference of insignificant factors to the well-established model. At the same time, it is cost-effective to reduce the number and improve the representativeness and accuracy of the financial indicators used to build the model. Firstly, it is necessary to observe that the premise of the indicator is distributed according to the normal distribution, therefore, to use the test of the normal distribution test to derive its distribution. If the results pass, i.e., they follow a normal distribution, then, the T-parameter test is used. However, if the results do not pass, i.e., they do not follow a normal distribution, then, the Mann-Whitney *U* non-parametric test is used.

Companies in the same detailed industry may have significant differences in key financial and tax indicators, due to the factors such as technology level and product type. Therefore, the early warning parameters should not be set simply by a 20% up or down fluctuation rate, but rather by standard deviation and dispersion coefficient to show the situation of the industry.


(1) $$\begin{array}{l} S=\sqrt{\frac{\overset{n}{\underset{i=1}{\sum }}{\left(X_{i}+\bar{X_{i}}\right)}^{2}}{n\;+\;1}} \end{array}$$

where, *S* is the standard deviation and is corrected when the value of S is large, and the corrected value is s.


(2) $$\begin{array}{l} s=\frac{\sqrt{\frac{\overset{n}{\underset{i=1}{\sum }}{\left(X_{i}+\bar{X_{i}}\right)}^{2}}{n\;+\;1}}}{n} \end{array}$$

(3) $$\begin{array}{l} \delta =\frac{s^{2}}{\bar{X_{i}}} \end{array}$$

The rate of change analysis also plays an important role in tax risk analysis, such as the change of sales rate expense to operate income ratio, which can reflect the change of enterprise operation (Asvija et al., 2021). When setting early warning indicators, only the fixed-value early warning can only screen single-enterprise enterprises with large fluctuations in the rate of change, which cannot well-reflect the industry law, and should compare the rate of change of its analysis object with the industry average rate of change.


(4) $$\begin{array}{l} \bar{X}=\sum xf^{2} \end{array}$$

The index of elasticity coefficient mainly reflects the relative magnitude of changes in two indexes with a logical correlation for single-family taxpayers. The same elasticity coefficient indexes of single-family enterprises in the same industry have certain rules, and the early warning parameter of the elasticity coefficient should reflect the normal amplitude range of changes of two indexes related to each other within a certain period for taxpayers in this industry. The reasonable setting of early warning of elasticity coefficient can effectively identify the abnormal data of single-family taxpayers within a certain period.


(5) $$\begin{array}{l} Z=\frac{x-x^{\prime }}{y-y^{\prime }}\;\cdot \;\frac{y}{x} \end{array}$$

(6) $$\begin{array}{l} E\left(Patent_{tt}\right)=e^{\left(\beta_{0}-\beta_{1}\ln\;TPatent_{tt}\right)} \end{array}$$

(7) $$\begin{array}{l} E\left(NPIP_{tt}\right)=e^{\left(\beta_{0}-\beta_{1}\ln\;TGS+\beta_{2}\;\ln\;TGS\right)} \end{array}$$

Specifically, in the field of tax service, there is a great information difference between the taxation department and the taxpayers. Taxation authorities have significant advantages in the information on tax laws and regulations, various tax policies, and tax procedures, while taxpayers are relatively disadvantaged in such information due to the time lag of policy implementation and the complexity and professionalism of tax policies. To reduce the information asymmetry in this regard, it must be realized by strengthening tax law publicity and training and counseling. Compared with taxation authorities, taxpayers have more specific information about their operation statuses, such as sales revenue, cost and expense, legal taxation situation, and demand for taxation service, and tax authorities are relatively disadvantaged, which is precisely the information needed to carry out accurate taxation service. To improve the quality and efficiency of tax services, information asymmetry must be reduced, and the collection, mining, and analysis of big data can help taxation authorities to improve the information asymmetry in this regard.

**Figure 4:**
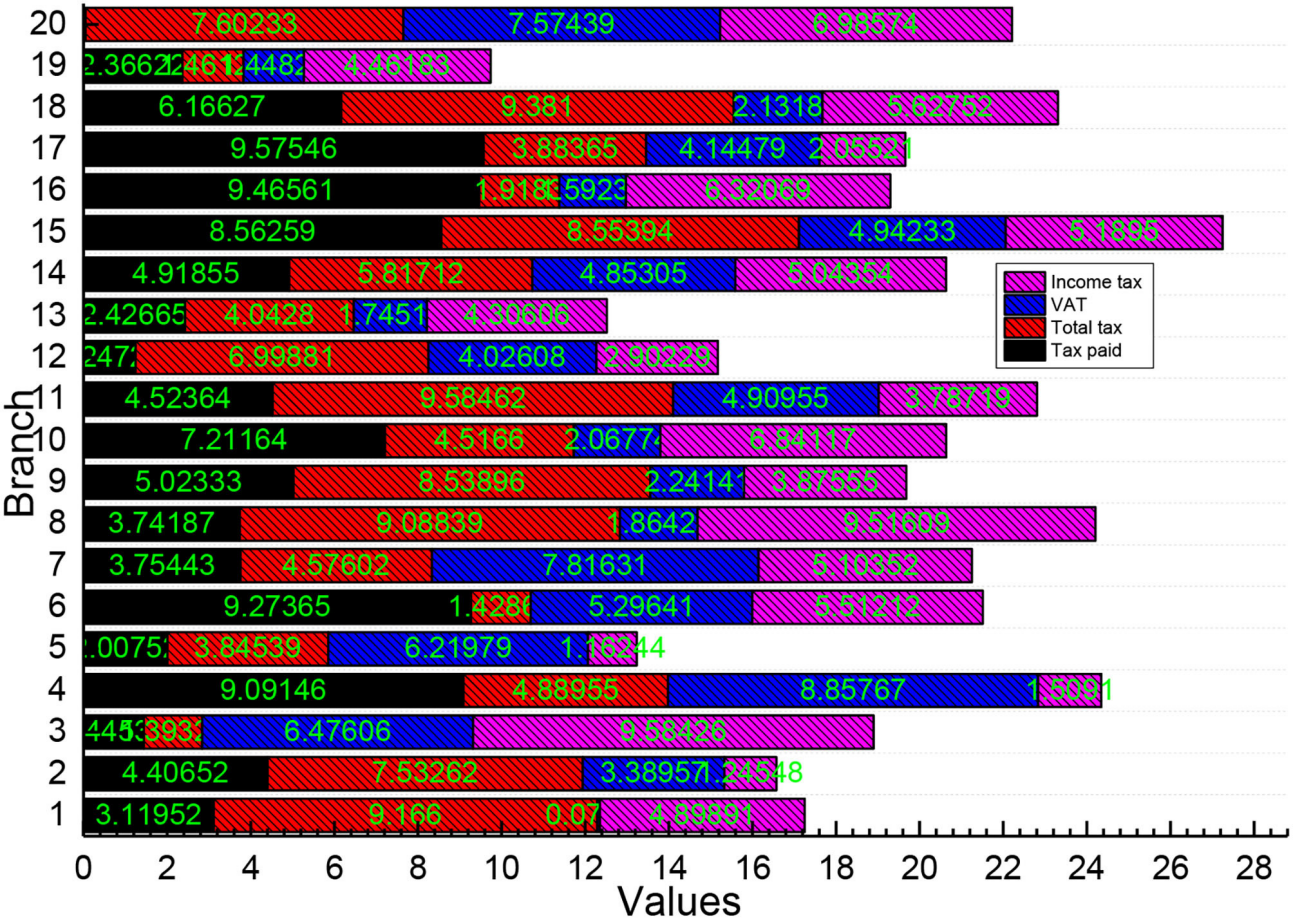
Statistics of tax assessment results.

Detailed analysis of taxpayers' past and present tax data and behavioral data can enhance the predictability of the tax service; the integration of data within the taxation system and all parties can discover more correlations between things and understand the tax service objects more multi-dimensionally; the mining and analysis processing of massive tax-related data can improve the scientific and accurate decision-making. Based on holding sufficient data information, the taxation department can better regulate and monitor the tax-related behaviors, analyze the changes in tax sources and provide the needed services, as shown in Figure 4.

The data collected by the current tax bureau rely on the tax registration and financial statements of enterprises, the key data reflecting the overall operation of the economy and the production status of specific taxpayers are not collected. It is difficult to ensure the accuracy, completeness, and timeliness of taxpayers' static data declarations. If Jinjiang Municipal Tax Bureau keeps to the surface data declared by taxpayers themselves, it will be difficult to identify the false information provided by taxpayers and process them instantly, and the data quality is prone to fall into a strange circle of endless problems and be difficult to correct. There is a lack of standardized data-sharing mechanisms. The sharing of tax-related information is especially important in the process of tax source management. Although relying on modern information technology can realize the information interaction of different departments, the current lack of rigid regulations of relevant laws makes different departments subjective and selective in providing data.

Taking the Market Supervision Administration as an example, the registration of new enterprises and the change of equity of old enterprises are the information that can be used for tax source management in the context of big data.

## Analysis of Results

### Results of Tax-Smart Risk Management Strategies for Big Data

**Figure 5:**
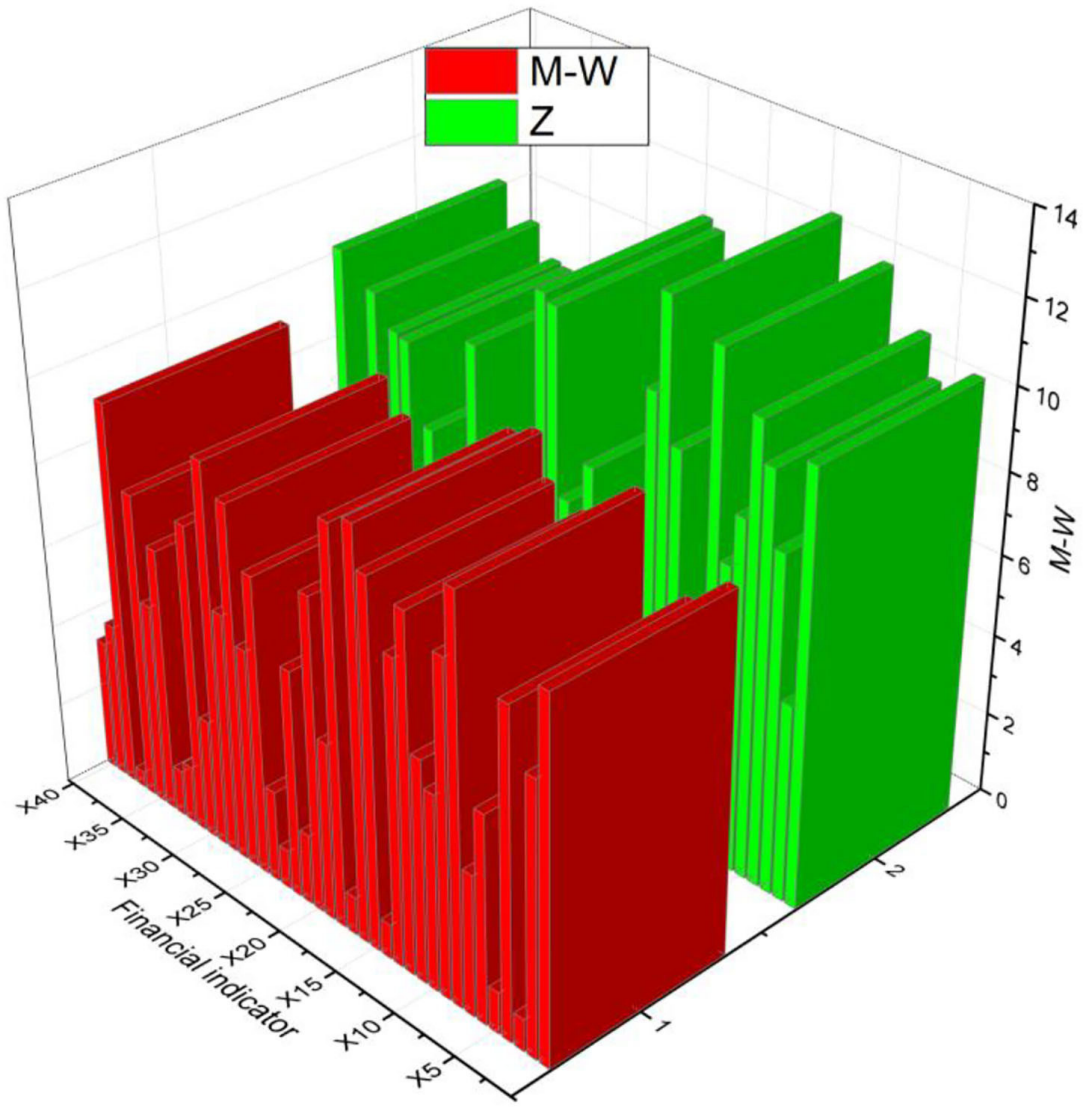
Non-parametric test results.

In the previous test, 49 indicators did not meet the normal distribution test. The Mann-Whitney *U* non-parametric analysis was performed to test the significance of these indicators to remove the non-significant indicators. The original hypothesis was first made that there is no significant difference between the financial indicators of the financial crisis and non-financial crisis. The significance criterion is 0.05, and the M-W and Sig values are calculated. If they are <0.05, the original hypothesis is not valid, i.e., there is a significant difference between the indicators of the sample groups; while if they are ≥0.05, the original hypothesis is valid, i.e., there is no significant difference between the indicators of the sample groups. The test results are shown in Tabs.4–6: X1, X3, X7, X11, ...... the significance level is 0.05, 18 indicators such as X1, X3, X7, X11, ......., etc. are less than 0.05, and the significance can be applied to the model; the remaining 31 indicators such as X2, X4, X5, ...... the remaining 31 indices such as X2, X4, X5, etc. are >0.05 did not pass the test and no signs should be discarded in the model, as shown in Figure 5.

**Figure 6:**
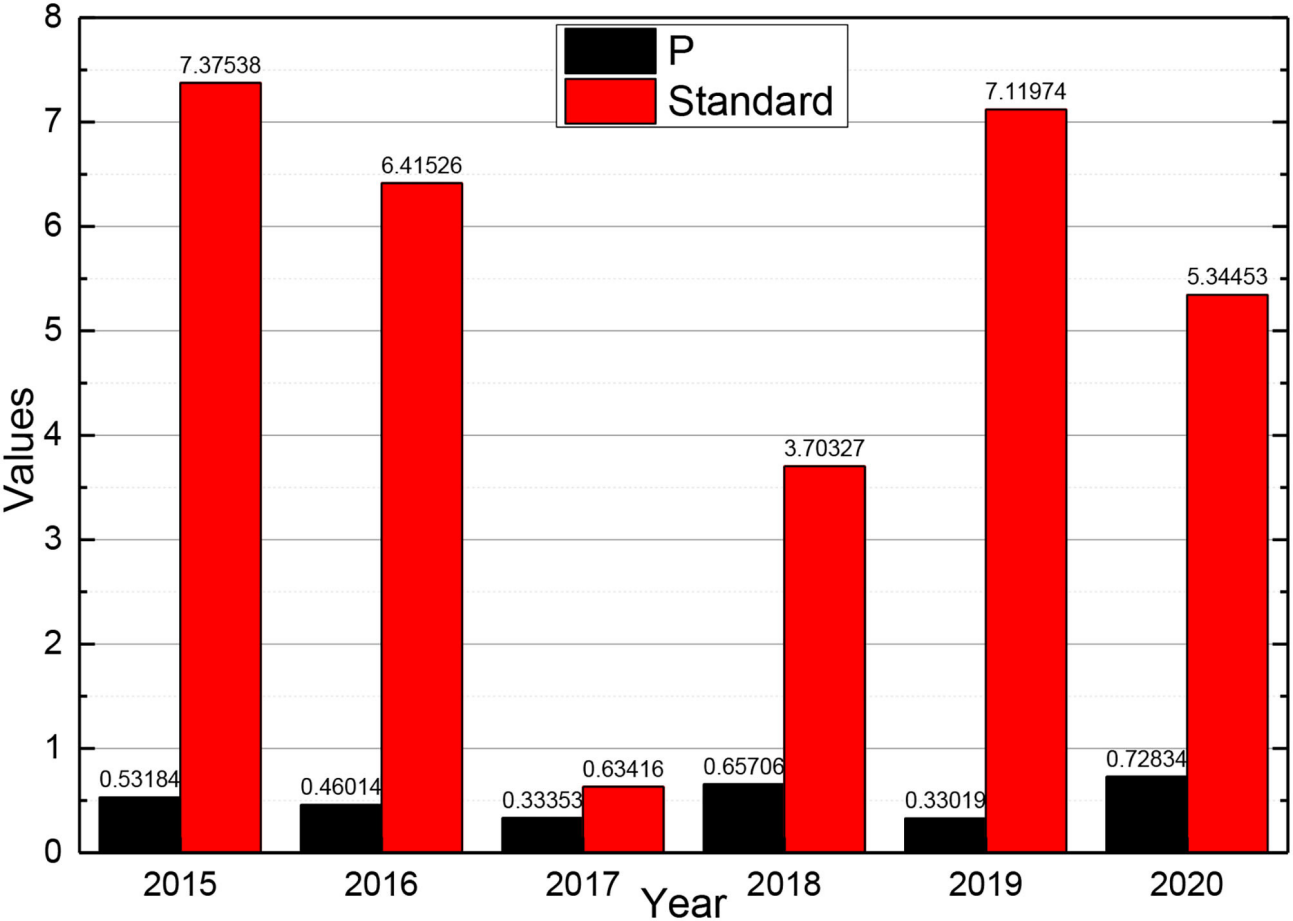
Change in *P*-value from 2015 to 2020.

The correct prediction rate of the test considering the big data model test is 85.3% by the test. With the introduction of big data indicators, the accuracy of the test for the ST sample and non-ST sample improves to 71.4 and 88.9%, respectively, and overall accuracy improves by 8.8%. This indicates that the addition of big data has a significant effect on the improvement of the financial crisis warning model. After establishing the industry model with the financial data of 60 new energy companies as the background, this paper crawled the shareholder comment data and conducted sentiment text analysis to derive non-financial indicators, i.e., big data indicators, to improve them. Then, the model was tested by selecting data from some companies in different years, which proves that the model is accurate and applicable to new energy companies to predict the financial crisis. After applying the means of big data, the comparison of its results can be concluded that the introduction of big data has a significant effect on the improvement of the accuracy of the financial crisis warning model. Based on the previous analysis of the financial indicators of M New Energy Company and the capture and operation of the big data indicators, the values of X7, X11, X20, X25, X34, X35, B, and C for each year are derived and substituted into the model that has been studied. Meanwhile, this paper is studying whether M New Energy Company will have a financial crisis in 2019 and 2020, and its financial data and big data indicators for 2017 and 2018 are to be studied. At the same time, the data of Company M from 2013 to 2020 are calculated so that they can be compared. Financial data and big data metrics for 2013–2018 will be calculated and extracted. After bringing each financial indicator into the model for calculation, the final *P*-value for 2015–2020 can be obtained, and the obtained *P*-value is compared with the criterion of 0.5, and the results are shown in Figure 6.

The financial data and big data indicators from 2013 to 2017 were calculated and extracted. After bringing each financial indicator into the model for calculation, it is possible to conclude whether there is a possibility of a financial crisis in 2015–2020. As given in the graph, the *P*-values for 2013–2016 are all < 0.5, and the fact that company did operate well from 2015 to 2018 and did not experience a financial crisis. This illustrates that the calculating results of the model are accurate. The final *P*-value for 2017 is calculated to be <0.5, indicating that the company's financial position is still normal in 2019. However, after calculating the data for 2018, the *P* > 0.5, which indicates that there is a possibility that the company will have a financial crisis in 2020. This has a great effect on the prevention of financial crises in the future. It can help the company to adjust its direction and change its strategy in the future management, find the problems and solve them in time. After the previous financial analysis of the company, we can also solve the problems that arise.

The analysis of the company's financial position shows that the inventory turnover ratio from 2015 to 2018 is substantially lower than before, especially in 2018, which is low in the industry. The company has a serious inventory pile-up, so it needs to strengthen the financial management system in terms of inventory turnover to turn over the inventory promptly. At the same time, capital turnover is also a very important factor, although the company's accounts receivable turnover is stable, it should continue to be maintained and controlled more. A better way is to manage inventories that take up a lot of capital, such as photovoltaic, separately from those that take up less capital, such as monocrystalline silicon wafers. It is also very important to revitalize assets promptly. The company's low turnover rate in 2018 can be changed by disposing of idle assets in total. This requires frequent attention to making an inventory of the company's assets.

### Results of Intelligent Risk Management Algorithms for Fiscal Taxes of Listing Companies

**Figure 7:**
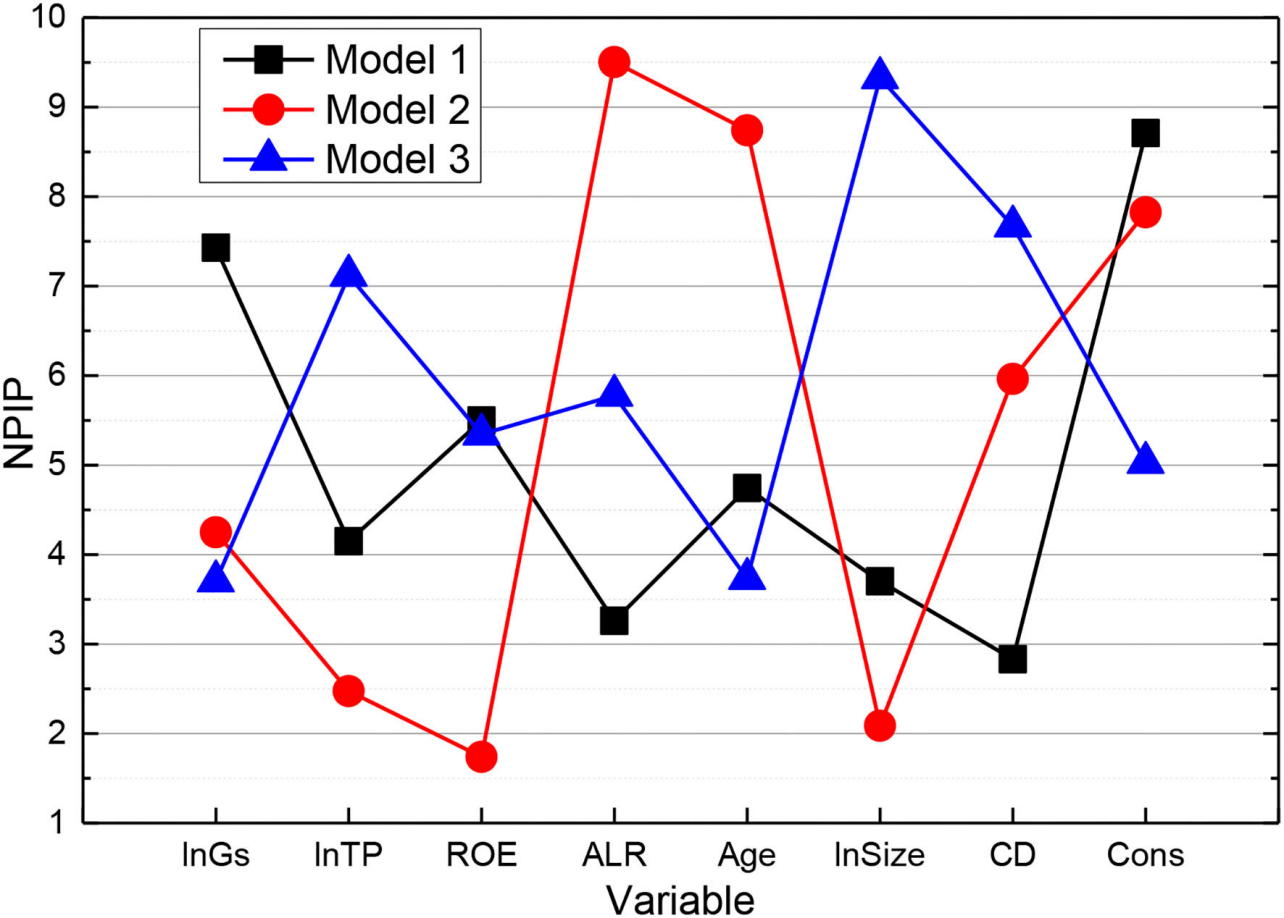
Negative binomial regression results.

As shown in Figure 7, Model 10 was developed to investigate the relationship between government subsidies and generic Import duty, VAT or rebate (IPRs) of digital culture-listed companies. For Model 10, the estimated parameters of government subsidies (GS) are positive and pass the 5% significance test, indicating that GS can stimulate the increase in the number of generic IPRs of digital culture listed companies, verifying Hypothesis 5. The data show that the subsidy parameter of GS is 0.1308, indicating that for every 1% government subsidy to digital culture listed companies, the number of listed companies' patent applications increases by 0.1308%. The estimated parameters of the control variables show that the estimated parameters of asset-liability ratio (ALR) and enterprise size (Size) are positive, and both pass the 1% significance test, indicating that ALR and Size positively contribute to the increase in the number of non-patents IPRs of digital culture listed companies. The estimated parameters of firm age (Age) and capital density (CD) are both negative and pass the significance tests of 10 and 1%, respectively, indicating that Age and CD not only do not have an incentive effect on generic IPRs of digital culture listed companies but also inhibit their enthusiasm to apply for generic IPRs.

Secondly, to investigate the relationship between tax incentives and generic IPRs of digital culture listed companies, model 11 is developed. from model 11, the estimated parameter of tax incentives (TP) is positive and passes the 10% significance test, indicating that TP can stimulate the increase in the number of generic IPRs of digital culture listed companies, which verifies hypothesis 5. The data show that the estimated parameter of TP is 0.2303, indicating that the number of listed companies' generic IPRs increases by 0.2303% for every 1% government tax concession for listed companies in digital culture. The introduction of control variables reveals that the estimated parameter of Asset-Liability Ratio (ALR) is positive and passes the 1% significance test, indicating that ALR has an incentive effect on the transformation of generic IPR achievements of digital culture listed companies. In addition, the estimated parameters of firm age (Age) and capital density (CD) are negative and pass the significance tests of 10 and 1%, respectively, indicating that Age and CD have a suppressive effect on the transformation of generic IPR results in digital culture listed companies.

**Figure 8:**
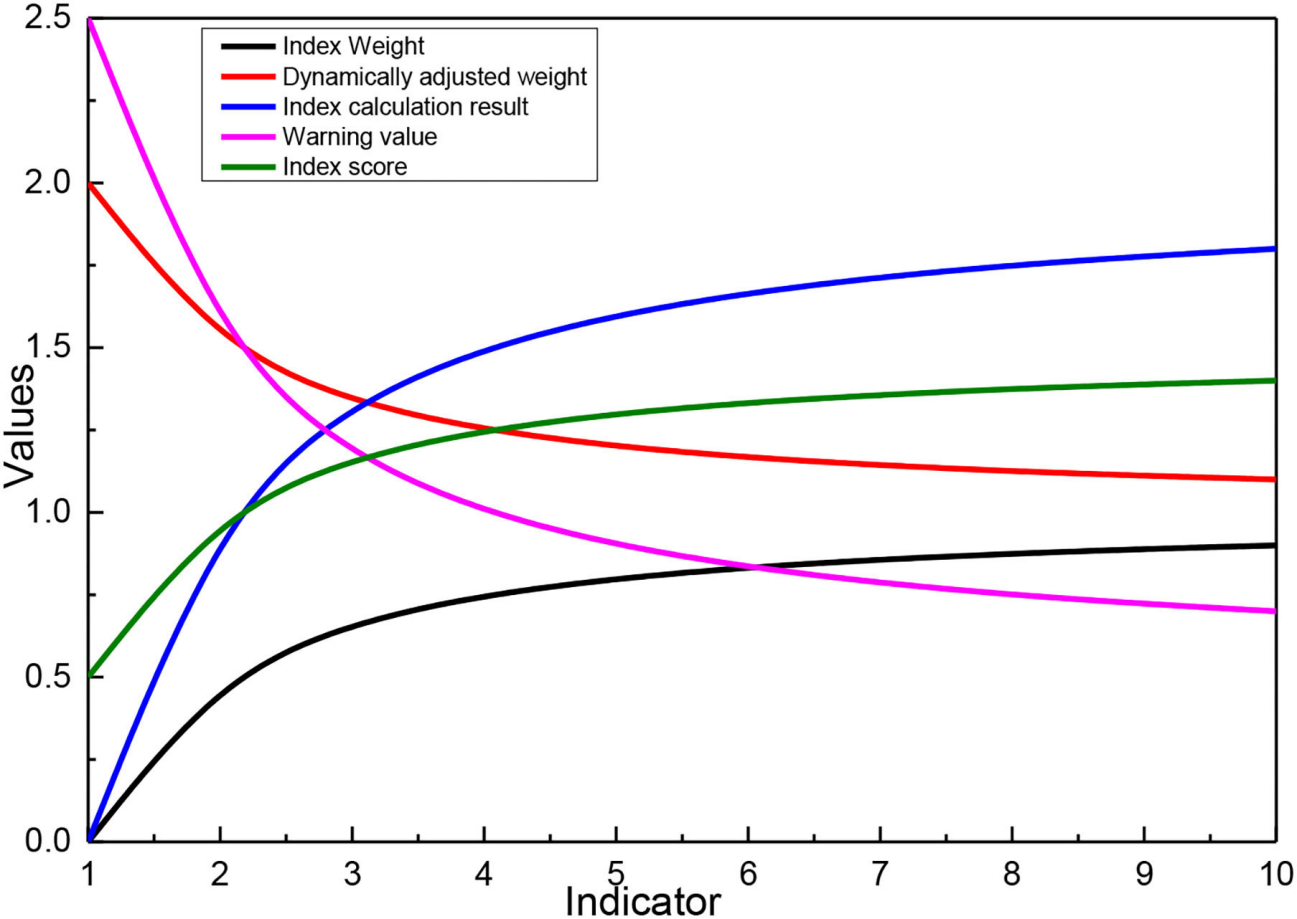
Optimized risk model identification results.

According to the optimization model for risk identification of listed companies, firstly, the dynamic indicators are verified. The input tax credit amount of agricultural products in the VAT return of listed companies is 0, so the risk of input tax ratio of agricultural products acquisition is involved in the calculation of the model score. The risk of abnormal loss is verified that the amount of specifically declared asset loss in line 9 of the schedule of asset loss (pre-tax deduction and tax adjustment schedule) of the annual income tax return is 0, so this indicator is excluded, and the indicator weight is assigned to other indicators according to the rules. Therefore, the listed company applies the dynamically adjusted indicator weighting calculation. Secondly, the weights of the model indicators need to be readjusted according to the results of the prior validation, and then the calculation results of each indicator are obtained according to the analysis and calculation on the BIEE database of JS provincial tax risk platform, and the warning value is set regarding the average value of the classification and grading industry. Then finally, the risk score of each indicator of the listed company is calculated, as given in Figure 8. The total score of the three indicators of sales expense rate, the change rate of comprehensive depreciation rate of fixed assets, and input tax ratio of agricultural products in the identification result of the optimized risk model is 23.66, among which the risk score of sales expense rate is higher and needs to be checked. By analyzing the correlation between the identification indicators and the tax assessment results, one of the two indicators of the original risk model is indirectly related to the assessed tax items, the total tax correlation score of the identification results is 1.

Two of the three indicators of the optimized risk model are directly related to the assessed tax items, and the total score of tax relevance of the identification result is 4. The experimental results show that the identification results of the optimized risk model are more directional than the original risk model. The risk model before and after optimization is analyzed for recognition effectiveness, and the tax relevance of each indicator is multiplied by its risk score to obtain the risk recognition effectiveness score of the indicator, and the recognition effectiveness score of the model was obtained after aggregation.

## Conclusion

With the launch of Golden Tax III, the database for tax risk management of listed companies by taxation authorities has been expanded and improved. This opens the era of big data tax management for listed companies. However, according to the actual situation, there are still many problems in the tax risk management of listed companies, especially the depth and breadth of the use of big data technology is not enough to meet the existing needs of tax risk management of listed companies. Firstly, we combed the literature related to tax risk management of listed companies and find that there are very few studies on listed companies with the background of big data. Through the literature combing, the theoretical foundation is laid for the subsequent research. Secondly, this work provides an in-depth analysis of the current situation and industry characteristics of tax risk management of listed companies, identifies the key points and difficulties of management, and summarizes the risks and problems of tax risk management of listed companies at this stage. Thirdly, this paper identifies the available scope of big data analysis for listed companies and optimizes and reconstructs the risk identification of listed companies' tax risk management based on it, which establishes a model framework for reference to carry out listed companies' tax risk management and improves the effectiveness of tax risk identification in the industry. Then, the effectiveness of the optimized model is verified through actual cases. The suggestions are made according to the actual situation, which provides an empirical reference for the subsequent development of tax risk management of listed companies.

Using big data thinking to improve tax risk identification capability. From a practical point of view, the first stage of tax risk identification is tax type-focused, the second stage of risk identification, which is the stage currently experienced, is industry-focused, and a future stage of risk identification will be big data-focused, based on tax type indicators and industry models, using big data technology to drive methodological innovation, not only limited to taxpayer tax and financial information, but also collecting taxpayers; adopt a model of co-management between the tax collector and the taxpayer, dynamically push the tax-related risks of taxpayers to the taxpayers, and change from being initiated by the tax authorities to risk prevention and control through tax-enterprise interaction in the whole process of tax collection and management.

## Data Availability Statement

The original contributions presented in the study are included in the article/supplementary material, further inquiries can be directed to the corresponding author.

## Author Contributions

XZ was responsible for designing the framework of the entire manuscript from topic selection to solution to experimental verification.

## Funding

This work was funded by 2020 Heilongjiang Province Philosophy and Social Science Research Planning Project (Youth Project), Performance evaluation and Enlightenment of Heilongjiang's tax reduction and reduction policy during the period of COVID-19 (No. 20JYC155).

## Conflict of Interest

The author declares that the research was conducted in the absence of any commercial or financial relationships that could be construed as a potential conflict of interest.

## Publisher's Note

All claims expressed in this article are solely those of the authors and do not necessarily represent those of their affiliated organizations, or those of the publisher, the editors and the reviewers. Any product that may be evaluated in this article, or claim that may be made by its manufacturer, is not guaranteed or endorsed by the publisher.
